# Mating rhythms of *Drosophila*: rescue of *tim*^01 ^mutants by *D. ananassae timeless*

**DOI:** 10.1186/1740-3391-4-4

**Published:** 2006-03-08

**Authors:** Izumi Nishinokubi, Masami Shimoda, Norio Ishida

**Affiliations:** 1 Clock Cell Biology, National Institute of Advanced Industrial Science and Technology, Institute of Molecular and Cell Biology, Tsukuba 305-8566, Japan; 2 National Institute of Agrobiological Sciences, Tsukuba 305-8634, Japan; 3 Institute of Applied Biochemistry, University of Tsukuba, Tsukuba 305-8576, Japan

## Abstract

**Background:**

It is reported that the circadian rhythms of female mating activity differ among *Drosophila *species and are controlled by an endogenous circadian clock. Here, we found that the mating rhythm of *D. ananassae *differed from that of *D. melanogaster*. Moreover, to evaluate the effect of clock gene products on mating activities, we examined the mating activity of *D. melanogaster timeless *(*tim*^01^) transgenic fly harboring heat-shock promotor driven-*D. ananassae timeless *(*tim*) gene (*hs*-AT *tim*^01^).

**Methods:**

Flies were maintained under light/dark (LD) cycles for several days and then they were transferred to constant dark (DD) conditions at 25°C. Transformant flies were heat-shocked for 30 min (PZT 10.5–11.0 or PZT 22.5–23.0; PZT means Projected Zeitgeber Time) at 37°C every day. Daily expressions of *D. ananassae *TIMELESS (TIM) protein in transgenic flies were measured by western blotting. To examine whether the timing of *D. ananassae *TIM protein induction by heat shock can change the patterns of the behavior activities of *D. melanogaster tim*^01 ^flies, we measured locomotor and mating activity rhythms under DD at 25°C ± 0.5°C except when heat shock was applied.

**Results:**

Heat shock applied at PZT 10.5–11.0 and at PZT 22.5–23.0 induced high TIM levels during subjective night and day, respectively, in *hs*-AT *tim*^01 ^flies. The locomotor rhythm of these flies was changed from diurnal to nocturnal by the timing of *D. ananassae *TIM induction. However, the mating rhythm of these flies could not be entrained by the timing of *D. ananassae *TIM induction.

**Conclusion:**

The pattern of mating activity rhythms of *D. ananassae *and of *D. melanogaster *differed. The mating activity rhythms of *D. melanogaster tim*^01 ^flies harboring *hs*-AT *tim *appeared after heat-shock but the pattern and phase differed from those of wild-type *D. ananassae *and *D. melanogaster*. Moreover, the mating rhythm of these flies could not be entrained by the timing of *D. ananassae *TIM induction although the locomotor rhythm of *hs*-AT *tim*^01 ^was changed from diurnal to nocturnal according to the timing of *D. ananassae *TIM induction. These data suggest that species-specific mating activities require output pathways different from those responsible for locomotor rhythms.

## Introduction

The behaviors of most organisms are subject to rhythms that are controlled by an endogenous circadian clock [1]. Clock genes including *period *(*per*), *timeless *(*tim*), *clock *(clk) and *cycle *(cyc) and their products constitute the core of the circadian mechanism. The sexual receptivity and reproductive behaviors of insects, for example courtship song, mating and ovipositor activities, are related to circadian mechanisms [2-6]. The locomotor activities of virgin queen ants are rhythmic whereas those of mated queens become arrhythmic when they lay eggs, but rhythmicity is restored after the eggs are deposited [7]. Clock genes of the melon fly may cause reproductive isolation through a change in the time of mating [8].

The rhythms of *Drosophila *mating behaviors are controlled by circadian clock genes and are especially attributed to the female clock [2]. Female circadian rhythm in mating activity is also species-specific, and this might constitute one source of the reproductive isolation that allows *Drosophila *to avoid sympatric hybridization. The mating behavior rhythms of *D. melanogaster *and *D. simulans *are different and in antiphase [2].

We also reported that the *tim *gene product is highly conserved between *D. melanogaster *and *D. ananassae*. *Tim *cDNA of *D. ananassae *could rescue the arrhythmic locomotor activity of the *D. melanogaster timeless *null mutant (*tim*^0^) [9].

The present study examines whether the mating activity rhythm of the *D. melanogaster tim*^01 ^mutant can also be rescued by introduction of the *D. ananassae tim *gene. We also determined whether the mating activity rhythm of transgenic flies carrying the *tim *gene from another species is affected by intrinsic locomotor rhythms of the original species.

## Materials and methods

### Animals

Flies grown on glucose-molasses-yeast-cornmeal were maintained at 25 ± 0.5°C under an LD cycle with lights on at 09:00 and lights off at 21:00. Transformant flies carrying *D. ananassae tim *cDNA were generated using P-element-mediated methods as described by Nishinokubi *et al*. [9].

### Mating activity assays

Virgin female and male flies maintained as described above for 7 days after eclosion were transferred to DD conditions for 2 days at 25°C. Transformant flies were heat-shocked for 30 min (PZT 10.5–11.0 or PZT 22.5–23.0; PZT means Projected Zeitgeber Time) at 37°C every day. We then analyzed the mating frequency of 9-day-old adult flies that were allowed to mate for 20 min as described by Sakai *et al*. [2,10]. In each experiment, the male flies were crossed with female flies of the same genotype.

### Locomotor assays

We tracked the movements of flies that were individually housed with medium, using infrared sensors and a *Drosophila *activity monitor (Trikinetics Inc, Waltham, MA) placed in an incubator under DD at 25°C ± 0.5°C except when heat shock was applied (30 min; PZT 10.5–11.0 or PZT 22.5–23.0 at 37°C daily). Signals from the sensors were summed every 30 min using a computer.

### Western blotting

Flies were entrained at 25°C ± 0.5°C under LD then transferred to DD for 2 days. Fly heads were collected on dry ice every 2 h (flies to which heat shock was applied at PZT 10.5–11.0) or 3 h (flies to which heat shock was applied at PZT 22.5–23.0) and Western blotted as described by Nishinokubi *et al*. [9].

## Results

**Figure 1:**
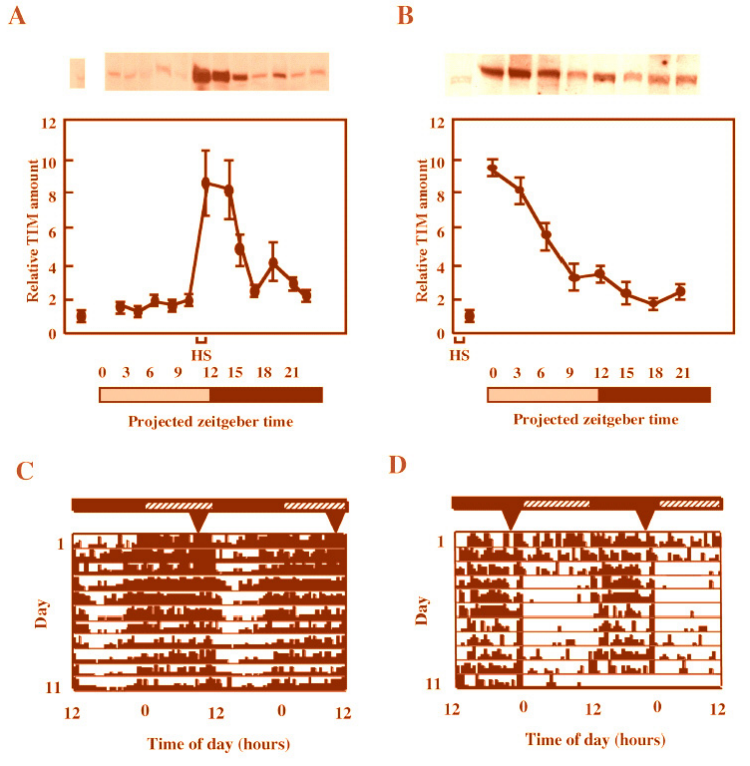
**TIM protein levels and locomotor activity rhythm in flies receiving heat shock under DD conditions**. *D. ananassae *TIM protein was induced by heat shock at PZT 10.5–11.0 (A) and at PZT 22.5–23.0 (B) in *w*, *tim*^01^, hs-*D. ananassae tim *flies. Heat shock at 37°C for 30 minutes every day rapidly induced TIM. The dotted lines depict data from non-heat-shocked control flies. (C) and (D) are representative double-plot actgrams of locomotor activitys of transgenic flies that were heat shocked at PZT 10.5–11.0 (C) and PZT 22.5–23.0 (D), respectively. Dark and shaded bars show subjective night and day under DD conditions. Heat shock was applied for 30 minutes at 37°C every day. Arrowheads indicate start point of heat shock.

In our previous report [9], *D. ananassae *TIM protein was functional for a clock component in *D. melanogaster tim*^01 ^flies when the timing of TIM induction was mimicked to wild type of *D. melanogaster*. In this study, we examined whether the timing of *D. ananassae *TIM protein induction by heat shock could change from diurnally active to nocturnally active for the locomotor rhythm of *D. melanogaster tim*^01 ^flies. Heat shock initially increased levels of TIM protein that decreased thereafter. Heat shock applied at PZT 10.5–11.0 and at PZT 22.5–23.0 induced high TIM levels during subjective night and day, respectively (Fig. 1A and 1B). We compared the locomotor activity rhythms of transgenic *tim*^01 ^flies carrying *D. ananassae tim *cDNA applying heat shock at different times of day. The arrhythmic flies heat shocked for 30 min at PZT 10.5–11.0 under DD became rhythmic and moved during subjective day ([9], Fig. 1C). On the other hand, flies that were heat shocked at PZT 22.5–23.0 became active during subjective night (Fig. 1D). These findings demonstrate that the locomotor behavior of *D. melanogaster tim*^01 ^flies harboring hs-*D. ananassae tim *became nocturnally active from the time of *D. ananassae *TIM induction.

**Figure 2:**
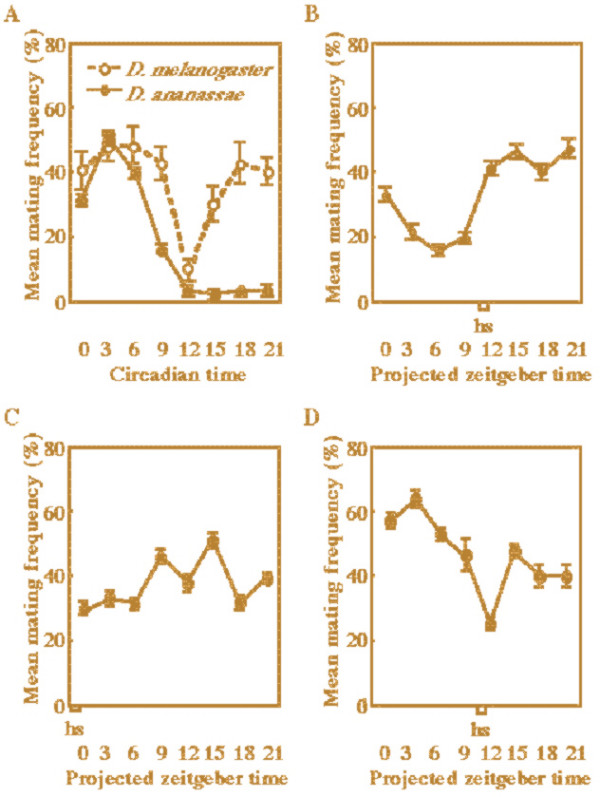
**Mating activities of flies at different times of day**. Error bars indicate SEM. Males and females were allowed to mate for 20 min. In each experiment, the male flies were crossed with female flies of the same genotype. (A) Mating activity rhythm of *D. melanogaster *Canton-S females crossed with *D. melanogaster *Canton-S males and in *D. ananassae *HW females crossed with *D. ananassae *HW males. (B) Mating activities in transgenic *w, tim*^01^, *hs-D. ananassae tim *flies that were heat shocked every day at PZT 10.5–11.0. (C) Mating activities in transgenic *w, tim*^01^, *hs-D. ananassae tim *flies that were heat shocked every day at PZT 22.5–23.0. (D) Mating activity rhythm of heat shocked *D. melanogaster *wild-type Canton-S female flies crossed with Canton-S males. Both males and females were applied heat shock at PZT 10.5–11.0.

The mating activities of *D. melanogaster *circadian clock mutants are arrhythmic, indicating that circadian clock genes control the rhythm of mating activity [2]. To understand the mating rhythm of *D. ananassae*, we first determined mating frequency at different times of the day. Interestingly, the mating rhythm of *D. ananassae *under DD differed from the mating rhythms of *D. melanogaster *and *D. simulans *(Fig. 2A and [2]). Most *D. ananassae *flies mated during subjective day rather than during subjective night (Fig. 2A).

**Figure 3:**
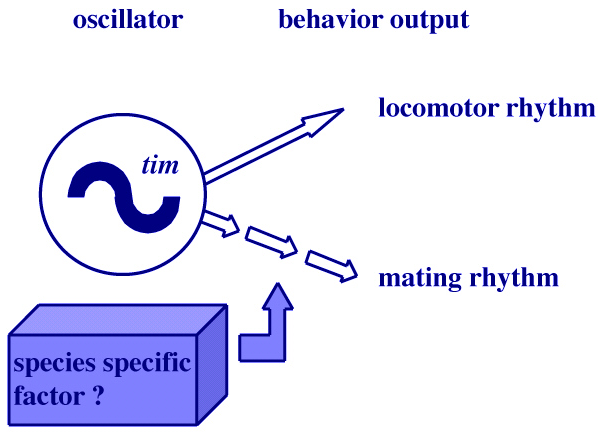
**An output model of the mating behavioral rhythm**. Outputs of behaviors are regulated by an intrinsic circadian oscillator, a component of which is the *tim *gene. The locomotor activity rhythm is directly correlated with the timing of TIM protein induction. On the other hand, the mating activity rhythm is regulated by different pathways. Unknown factors may affect the profile of the mating activity rhythm.

We then measured mating activity in transgenic *tim*^01 ^flies carrying *D. ananassae tim *cDNA to determine whether mating activity can also be rescued and whether it is related to levels of the circadian gene product TIM. The TIM protein levels in wild-type *D. ananassae *flies were initially higher during subjective night than during subjective day, like those of *D. melanogaster *(data not shown). Therefore, we exposed *D. melanogaster tim*^01 ^flies harboring hs-*D. ananassae tim *to heat shock at PZT 10.5–11.0 and measured their mating activity. The mating activity of heat shocked transgenic *tim*^01 ^flies was rhythmic and higher during subjective night than during subjective day (Fig. 2B). When the transgenic flies were heat shocked at PZT 22.5–23.0, a bi-phasic pattern of mating activity appeared around PZT 12 (Fig. 2C). These profiles of mating activity rhythm of heat shocked transgenic *tim*^01 ^flies differed from both *D. ananassae *and the background *D. melanogaster *(Fig. 2A). These data indicated that TIM is not enough for the generation of species-specific mating rhythm and suggested that species-specific mating rhythms require more factors or different pathways than those required for the locomotor activity rhythm (Fig. 3).

To investigate the effect of heat-shock on the mating activity rhythm, we measured mating activity rhythm of *D. melanogaster *wild-type flies under heat shock conditions. Even if flies were heat shocked at PZT 10.5–11.0, the profile of mating activity rhythm of these flies was not significantly different from that of wild-type, although mating rates of wild-types under heat shock conditions slightly increased (Figs. 2A and 2D). This result indicated that heat shock itself did not significantly affect the profiles of the mating activity rhythm.

## Discussion

We previously reported that the mating activity and locomotor activity of *D. melanogaster tim*^01 ^mutant flies are arrhythmic [2]. The present study showed that *D. melanogaster tim*^01 ^flies harboring *D. ananassae tim *cDNA had mating activity rhythms, but the profile differed from both *D. melanogaster *and *D. ananassae*. These rhythms were not bimodal as observed in *D. melanogaster *and their peaks were largely delayed relative to *D. ananassae *(Fig. 2A–C). The circadian clock gene, *period*, plays a role in the mating rhythms of flies [6]. The mating peak of the *D. pseudoobscura *transformant line that expresses *D. pseudoobscura per *fused to the *D. melanogaster per *promoter was later than that of *D. pseudoobscura *[6]. These results supported the notion that the *period *gene plays a role in temporal reproductive isolation between populations of closely related species [6] or within a species [8] by changing the timing of mating behavior. The present data suggested that *tim *might also be a putative speciation gene like *period*. Our previous report showed that the homology of full length TIM protein with *D. ananassae *and *D. melanogaster *was 85.9%. In particular, the PER interaction domains and NLS were highly conserved (PER interaction domain-1; 96.0%, PER interaction domain-2; 95.0%, NLS; 100%) [9]. Other regions with lower homology with *D. melanogaster *TIM (e.g. CLD; 70.8%) may be important for the establishment of species-specific behavioral rhythms such as the mating activity rhythm.

Our data show that heat shocked wild type flies mate well (Fig. 2D). The increase in temperature may enhance the volatility of gaseous mating factors (pheromones) and therefore increase mating activity.

Male courtship songs differ among *Drosophila *species and contribute to sympatric speciation [11]. Females discriminate *D. ananassae *and its sibling species, *D. pallidosa*, according to male courtship songs, and the loci of sexual isolation appear to map near the Delta locus of the second chromosome [12]. *D. melanogaster *and *D. simulans *are sibling and sympatric species that have not been isolated by geographical location. However, their mating behavior rhythms are in antiphase. During frequent *D. melanogaster *mating, the activity of *D. simulans *mating is lower, but the activity becomes higher when *D. melanogaster *mates infrequently [2]. The mating behavior rhythm of *D. ananassae *is unique among those of *D. melanogaster *and other species (Fig. 2A, [2,6]). Our data support the notion that the species-specific timing of mating behavior (including differences in male courtship songs) plays a role in reproductive isolation for sympatric speciation.

The present study showed that the locomotor activity rhythm of *tim *transformant flies carrying *D. ananassae tim *cDNA can be entrained by the timing of heat shock application (Figs. 1A–1D). The locomotor activity level of *D. melanogaster *is higher during subjective day, and the TIM level is higher during subjective night [13]. Our transformant flies were similarly active during subjective day when heat shock was applied during subjective night (Figs. 1A and 1C). On the contrary, when flies were heat-shocked to increase TIM levels during subjective day, their activity levels were higher during subjective night (Figs. 1B and 1D). These results demonstrated a close correlation between the phases of the locomotor activity rhythm and the timing of TIM induction. On the other hand, the phase of the mating activity rhythm appears not to be correlated with that of the timing of TIM induction because the mating pattern of our transgenic flies at different times of TIM induction (Figs. 2B and 2C) and wild-type flies (Fig. 2A) differed. Our data strongly suggest that the rhythms of locomotor activity and mating behavior have different output pathways from the central circadian system (Fig. 3). Tauber *et al*. also suggested that periods of locomotor activity are not causally related to mating behavior, although the two rhythms may be manifestations of the same central oscillator [6]. Thus, we propose that the pathways of the mating activity rhythm are more molecularly complex than those of the locomotor activity rhythm.

Locomotor activity is related to mating or sexual receptivity in many insects [14]. For example, virgin ant queens have a circadian locomotor activity rhythm whereas mated queens laying eggs do not show circadian and their activity levels are much lower than those of virgin females. Mated queens that have stopped laying eggs resume circadian locomotor rhythm [7]. Female German cockroaches that display higher locomotor activity are sexually receptive and such activity is reduced after mating, suggesting that the female locomotor activity is primarily associated with finding a mate. [15]. Most investigations addressing the relationship between locomotor activity and mating or sexual activity have been conducted after the flies had mated (post-mating). However, considering these data, further study is required to clarify the pre-mating mechanism, including the mating behavior rhythm, to determine the molecular mechanism of speciation.

## Competing interests

The author(s) declare that they have no competing interests.

## Authors' contributions

IN participated in data collection and data analysis and drafted the manuscript. MS helped produce the transgenic flies and supervised the study. NI directed the study. All authors read and approved the final version of the article.
